# Postpneumonectomy Syndrome: Follow-Up, Treatment, and Prevention

**DOI:** 10.1016/j.atssr.2025.12.017

**Published:** 2026-01-12

**Authors:** Domenico Galetta, Maria Serra

**Affiliations:** Division of Thoracic Surgery, San Giovanni Bosco Hospital, Asl Città di Torino, Piazza del Donatore di Sangue, 3, 10154 Turin, Italy; ASST Santi Paolo e Carlo, Milan, Italy

To the Editor

We recently read with great interest the article by Holmes and associates^1^ in the *Annals of Thoracic Surgery Short Reports*. This study reports the case of a patient with atypical presentation of a postpneumonectomy syndrome (PPS) caused by the cardiac-related anatomic changes that initially were manifested 6 months after surgery (shortness of breath, worsening dyspnea on exertion, and tachycardia with exercise). A chest computed tomography (CT) scan performed 12 months after surgery revealed elevation of the right diaphragm and subsequent nearly complete occupation of the right side of the chest by the liver, which resulted in a “mass effect” on the right side of the heart. The authors have corrected the anatomic abnormality and have resolved the symptoms, performing diaphragmatic plication and intrathoracic placement of an intrathoracic tissue expander.

The reported case was unique for the pathophysiologic features, and the authors should be congratulated for the success of the treatment.

We have some comments and questions. A systematic review on PPS reported that the median interval between the original pneumonectomy and the time of attempted correction for PPS was 2.5 years with a mean interval of 5.1 years.^2^^,^^3^ In the reported case, the diaphragm elevation was expected after phrenic nerve resection. Respiratory and cardiac symptoms initially appeared 6 months after operation, and no evidence of chest CT scan was reported. A short follow-up could have anticipated the problem. The authors should clarify what their follow-up management is for patients who underwent pneumonectomy and also what they think about the “preventive treatment” of PPS, which in the reported case was highly likely to occur because of the phrenic nerve resection.

The authors used intrathoracic saline tissue expander to fill the right-sided chest cavity. They did not clarify whether they used a prepackaged saline-filled prosthesis or a saline-filled prosthesis equipped with a subcutaneously placed injection port to adjust prosthesis size by adding or removing saline.

Currently, the insertion of saline-filled prostheses in the empty hemithorax, the most common approach, is considered safe for the treatment of PPS.^4^^,^^5^ We believe that the intrathoracic insertion of saline tissue expander remains the most effective procedure because it gradually rebalances the mediastinum above all when the external injection of saline solutions is used.

Although PPS is a rare event, we suggest performing a short follow-up (each 4 months) with clinical evaluation and chest CT scan for the first 2 years after pneumonectomy, in particular for those patients who had phrenic nerve damage, to prevent fatal events. For the treatment of PPS, we recommend the use of saline-filled prostheses in place of gel-filled prostheses for the high risk of complications, such as immune-related or connective tissue disorders. Prevention of PPS with saline tissue expander is a matter of debate.
